# Evaluation of acute flaccid paralysis surveillance performance before and during the 2014-2015 Ebola virus disease outbreak in Guinea and Liberia

**DOI:** 10.11604/pamj.2023.45.190.21480

**Published:** 2023-08-30

**Authors:** Grace Umutesi, Troy D Moon, Jeevan Kumar Makam, Fabien Diomande, Charlotte Buehler Cherry, Roland NO Tuopileyi II, Wambai Zakari, Allen Scott Craig

**Affiliations:** 1 Vanderbilt Institute for Global Health, Nashville, Tennessee, United States of America,; 2 Centers for Disease Control and Prevention, Atlanta, Georgia, United States of America; 3 World Health Organization, Monrovia, Liberia

**Keywords:** Polio, Ebola, surveillance, health systems

## Abstract

**Introduction:**

the number of wild poliomyelitis cases, worldwide, dropped from 350,000 cases in 1988 to 33 in 2018. Acute flaccid paralysis (AFP) surveillance is a key strategy toward achieving global polio eradication. The 2014 Ebola virus disease (EVD) epidemic in West Africa infected over 28,000 people and had devastating effects on health systems in Guinea, Liberia, and Sierra Leone. We sought to assess the effects of the 2014 Ebola outbreak on AFP surveillance in Guinea and Liberia.

**Methods:**

a retrospective cross-sectional analysis was performed for Guinea and Liberia to evaluate EVD´s impact on World Health Organization (WHO) AFP surveillance performance indicators during 2012-2015.

**Results:**

both Guinea and Liberia met the WHO target non-polio AFP incidence rate nationally, and generally sub-nationally, prior to the EVD outbreak; rates decreased substantially during the outbreak in seven of eight regions in Guinea and 11 of 15 counties in Liberia. Throughout the study period, both Guinea and Liberia attained appropriate overall targets nationally for “notification” and “stool adequacy” indicators, but each country experienced periods of poor regional/county-specific indicator performance.

**Conclusion:**

these findings mirrored the negative effect of the Ebola outbreak on polio elimination activities in both countries and highlights the need to reinforce this surveillance system during times of crisis.

## Introduction

In 1988, the World Health Assembly adopted a resolution to eradicate polio worldwide by 2000 and launched the Global Polio Eradication Initiative (GPEI) [1]. At that time, wild poliovirus (WPV) was circulating in more than 125 countries [2]. Tremendous progress toward eradication has been achieved, with the number of WPV cases worldwide decreasing from an estimated 350,000 in 1988 to 33 confirmed in 2018 [3]. Of the three WPV serotypes, type 1 remains the only confirmed circulating serotype. In 2020, Nigeria and the WHO Africa Region were declared free of wild poliovirus transmission while confirmed WPV transmission continued without interruption in only two countries: Afghanistan where 56 WPV cases were reported and Pakistan with 84 reported cases [3,4]. The World Health Organization (WHO) recommends the following strategies to eradicate polio: 1) attain high routine immunization coverage; 2) conduct supplementary immunization activities (SIAs) such as national immunization days (NIDs) or sub-national immunization days (SNIDs); 3) implement acute flaccid paralysis (AFP) surveillance with virologic testing of stool specimens; and 4) conduct mop-up immunization campaigns in defined areas where poliovirus is known or suspected to be circulating to ensure that every child is vaccinated [5,6]. AFP surveillance aims to effectively detect and investigate every newly paralyzed child to determine if poliovirus is the cause of the paralysis. Timely detection of AFP cases has been critical for enabling ministries of health to implement appropriate response activities and immunization campaigns with support from GPEI partners if WPV importation was to occur [7-9].

Oral poliovirus vaccine (OPV) containing live, attenuated (Sabin) viruses has been the choice for vaccination in developing countries because of its ease of administration, its ability to induce intestinal immunity, and it´s significantly lower cost [10]. However, it also comes with a rare risk of paralysis due to occurrence of vaccine-associated paralytic poliomyelitis, the emergence of vaccine-derived polioviruses (VDPV) or circulating vaccine-derived polioviruses (cVDPVs) in under-immunized populations [11,12]. To completely eradicate poliovirus disease, all OPV use will be stopped after WPV eradication. Because >90% of cVDPVs from 2000-2015 were type 2 (cVDPV2), and because WPV type 2 was declared eradicated in 2015, polio programs worldwide stopped the use of OPV type 2 (trivalent OPV, types 1, 2 and 3) by May 2016 and began using bivalent OPV (bOPV) (types 1 and 3) [13,14]. A variety of different crises, from political conflict and civil war to natural or other manmade disasters, have presented obstacles to the full implementation of polio eradication strategies and other public health initiatives [15-17]. In West Africa, the most recent crisis to dramatically disrupt public health systems was the Ebola Virus Disease (EVD) outbreak of 2014-2015. Guinea, Sierra Leone, and Liberia were the most affected countries, where approximately 28,000 EVD cases and more than 11,000 EVD-related deaths occurred [18-21]. The first EVD case was confirmed in Guinea in March 2014, and by May 2014 EVD cases were confirmed in Liberia and Sierra Leone [22,23].

This EVD epidemic disproportionately affected health systems, with health care workers being 21-32 times more likely to be infected than members of the general adult population [24]. In these three countries, 881 health care workers were infected, of whom 513 died, accounting for an overall reduction in the health care workforce of 8% in Liberia, 7% in Sierra Leone, and 1% in Guinea [25,26]. Remaining health care resources were directed toward EVD outbreak surveillance and control activities. Overall access to health care services decreased considerably in Liberia during the outbreak, resulting in increased mortality rates for malaria, HIV/AIDS, and TB [27,28]. Studies examining the impact of the Ebola outbreak on health systems in Sierra Leone showed that the outbreak stretched the capacity of human resources, leading to a reduction of routine vaccination coverage and surveillance activities [29,30]. WHO/UNICEF estimates of national polio immunization coverage (OPV3) for Guinea decreased from 56% in 2012 and 49% in 2013 to 42% in 2014 and 45% in 2015. Similarly, the estimated national coverage in Liberia decreased from 80% in 2012 and 75% in 2013 to 49% in 2014 and 52% in 2015 [31]. To evaluate the impact of the EVD outbreak on AFP surveillance performance in Guinea and Liberia, standard WHO AFP surveillance performance indicators were analyzed for the years immediately before and during the EVD outbreak [9].

## Methods

A retrospective cross-sectional analysis was performed using 2012-2015 AFP surveillance data routinely collected by the Ministries of Health of Guinea and Liberia and their data by sub-national area on the number of children aged < 15 years (the agreed upon age for polio surveillance). World Bank estimated population growth estimates for each country were used to retrospectively calculate the estimated population of children aged < 15 years [32]. For Guinea, the 2015 population data for children aged < 15 years and the 2015 estimated population growth of 2.7% were used to calculate the estimated size of the population of children < 15 years of age for the three previous years [32]. For Liberia, the 2016 population data for children aged < 15 years and the yearly estimated population growth of 2.4% were used [32]. Selected AFP surveillance indicators were calculated according to the WHO Regional Office for Africa guidelines [6] at the first sub-national administrative level; this corresponds to the “region” in Guinea and the “county” in Liberia. Guinea has eight regions and Liberia has 15 counties. Data purged of personal identifiers collected at these geographic levels for each country were used to assess the AFP surveillance indicators for the pre-EVD period (2012-2013) and during the outbreak (2014-2015).

### Case classification and timeline

AFP cases are classified as WPV (1, 2, or 3) or VDPV (1, 2, or 3) if stool specimens were culture positive for these polioviruses. If not, and two specimens were collected within 14 days from onset of paralysis and arrived in good condition at the WHO-accredited laboratory (“adequate” specimens), the case was discarded as a non-polio AFP case (NP-AFP), that is, as a paralytic illness due to one of various other background causes. If either of the components of specimen collection and handling was not met (“inadequate” specimens), the surveillance officer revisited the case at approximately 60 days to see if the case had any residual paralysis. If there was no residual paralysis, the case was discarded as NP-AFP. If there was evidence of residual paralysis, or if the child died or was lost to follow-up, the Polio Expert Committee reviewed the detailed investigation and epidemiological data of the case and classified it as either polio-compatible or discarded NP-AFP [5,6]. Guinea and Liberia use the WHO-recommended AFP case definition for polio surveillance among individuals aged < 15 years identified with symptoms consistent with AFP (new onset “floppy” weakness or paralysis) and persons of any other age with a paralytic illness in which polio is suspected by the clinician [5,6]. To evaluate the impact that EVD may have had on AFP surveillance performance, selected standard annual indicators were calculated for these countries for 2012 and 2013 (the “pre-Ebola” period) and 2014 and 2015 (the “Ebola” period) based on the date of onset of paralysis.

Three standard WHO AFP surveillance performance indicators were examined to evaluate the yearly performance of Guinea´s and Liberia's surveillance systems during the pre-Ebola and the Ebola outbreak periods. The first indicator assessed was the NP-AFP rate, defined as the total number of NP-AFP cases identified in a year in the population aged < 15 years per 100,000 population; the WHO target for Africa is at least two NP-AFP cases per 100,000 population. NP-AFP cases among those 15 years of age or older were excluded from calculation of the NP-AFP rate. The second indicator was notification, which refers to the proportion of AFP cases that were reported to the health system within 7 days of paralysis onset; the target is at least 80%. Lastly, the indicator for stool adequacy assessed the proportion of AFP cases that had two stool specimens collected within 14 days of paralysis onset that were judged to be in good condition upon arrival at the laboratory. The target for this indicator is at least 80% [5,6]. Cases that had missing data for these last two specific indicators (often, stool condition was missing) were removed from the numerators and denominators. Epi Info7 (Centers for Disease Control and Prevention, Atlanta, GA, USA) was used to perform descriptive statistics and to calculate proportions of targets met in the AFP surveillance system.

### Ethical consideration

The Institutional Review Board from Vanderbilt University and the Center for Global Health´s Office of the Associate Director for Science, Centers for Disease Control and Prevention, reviewed the study protocol, and exemptions were granted because this work was considered a public health program evaluation.

## Results

### AFP case notification and final classification

In Guinea, the number of AFP cases reported annually was 187 (182 aged < 15 years) in 2012, 224 (223 aged < 15 years) in 2013, 146 (145 aged < 15 years) in 2014, and 145 (143 aged < 15 years) in 2015. In 2012 and 2013, all AFP cases among children aged < 15 years were subsequently classified as NP-AFP (Table 1). In 2014, VDPV type 2 (VDPV2) was isolated from the stool of a 3-year-old boy from Kankan region who had paralysis onset on August 30, 2014; the remaining 145 cases were classified as NP-AFP. Of the 145 AFP cases reported in 2015, seven cases in Kankan region were confirmed as VDPV2 with onset dates from July to December 2015, with confirmation of the first 2015 case in September. Because VDPV2 isolates from all 2015 cases were genetically related to the virus isolated from the VDPV2 case in 2014, all eight of these cases during 2014-2015 were classified as cVPDV2 from a single emergence. The 2014 cVDPV2 case and six of the 2015 cVDPV2 cases occurred in the district of Siguiri and one occurred in the district of Kankan. In 2015, one AFP case from the neighboring region of Farannah was classified as compatible. The 137 remaining cases were classified as NP-AFP. In Liberia, 56 (54 aged < 15 years) AFP cases were reported in 2012, 50 (48 aged < 15 years) in 2013, 23 (22 aged < 15 years) in 2014, and 22 (22 aged < 15 years) in 2015. All cases were classified as NP-AFP (Table 1).

**Table 1:** acute flaccid paralysis (AFP) cases and non-polio acute flaccid paralysis (NP-AFP) Cases < 15 years, Guinea and Liberia, by region/county (2012-2015)

	2012			2013			2014			2015		
	Pop <15‡	AFP Cases	NP-AFP	Pop <15	AFP Cases	NP-AFP	Pop <15	AFP Cases	NP-AFP	Pop <15	AFP Cases	NP- AFP
GUINEA*												
Boke	459,152	25	23	471,549	19	19	484,282	24	24	497,357	18	18
Conakry	708,129	22	22	727,249	40	40	746,883	20	19	767,050	14	14
Faranah	400,258	19	18	411,066	22	22	422,164	13	13	433,563	14	13
Kankan	843,342	24	24	866,111	25	24	889,495	24	23 α	913,513	29	21 β
Kindia	661,987	26	25	679,861	27	27	698,217	13	13	717,069	10	10
Labe	422,754	26	25	434,169	24	24	445,891	18	18	457,930	17	16
Mamou	310,837	9	9	319,229	20	20	327,848	14	14	336,700	18	18
Nzerekore	706,312	36	36	725,382	47	47	744,967	20	20	765,082	25	25
**National**	4,512,771	187	182	4,634,616	224Þ	223	4,759,747	146#	144 α	4,888,264	145	135 β
**LIBERIA**†												
Bomi	40,655	7	7	41,630	3	3	42,630	2	2	43,653	1	1
Bong	161,172	3	3	165,040	4	4	169,001	2	2	173,057	0	0
Gbarpolu	40,301	1	1	41,269	1	1	42,259	2	2	43,273	1	1
Grand Bassa	107,145	3	3	109,717	1	1	112,350	1	1	115,046	3	3
Grand Cp Mt	61,416	1	1	62,890	2	2	64,399	2	2	65,945	0	0
Grand Gedeh	60,538	1	1	61,990	1	1	63,478	1	1	65,002	2	2
Grand Kru	27,990	2	2	28,661	1	1	29,349	1	1	30,054	1	1
Lofa	133,808	4	4	137,020	3	3	140,308	0	0	143,676	0	0
Margibi	101,456	5	4	103,891	6	6	106,385	2	1	108,938	0	0
Maryland	65,699	2	2	67,276	1	1	68,891	1	1	70,544	2	2
Montserrado	540,448	14	13	553,419	13	11	566,701	2	2	580,302	5	5
Nimba	223,298	4	4	228,657	6	6	234,145	3	3	239,765	5	5
Rivercess	32,279	3	3	33,054	1	1	33,847	1	1	34,660	0	0
River-Gee	34,561	2	2	35,390	2	2	36,239	1	1	37,109	1	1
Sinoe	49,486	4	4	50,673	5	5	51,889	2	2	53,135	1	1
**National**	1,680,252	56	54	1,720,578	50	48	1,761,872	23	22	1,804,157	22	22

* Estimated population based on a 2.7% growth/ year (World Bank) (32). † Estimated population based on a 2.4% growth/ year (World Bank) (32). ‡Population aged <15 years. Þ91cases missing a stool condition. #39 cases missing a stool condition. α1 circulating vaccine-derived poliovirus type 2 (cVDPV2) case detected in 2014. β7 circulating vaccine-derived poliovirus type 2 (cVDPV2) cases detected in 2015. AFP cases - all acute flaccid paralysis cases. NP-AFP: number of non-polio acute flaccid paralysis cases aged < 15 years. Cases with missing data were deducted from the numerators and denominators for the calculation of the indicators concerned.

### NP-AFP rates

**Figure 1:**
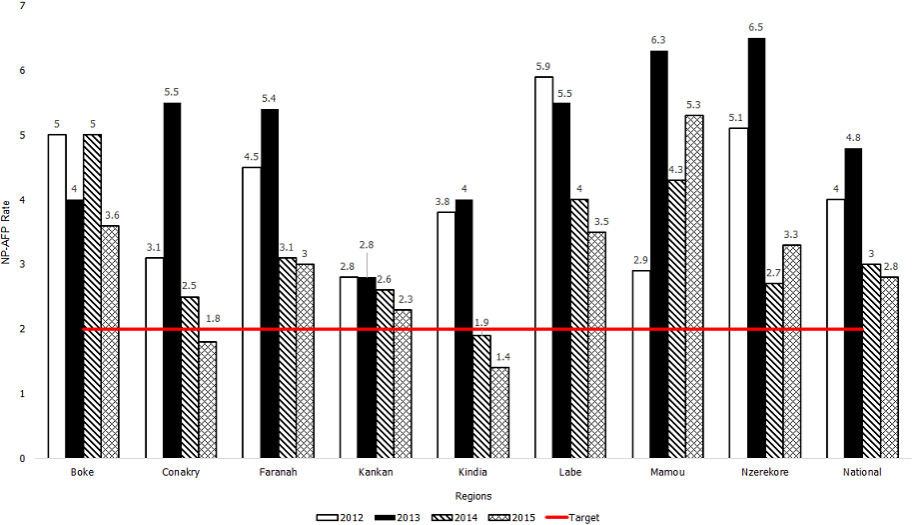
acute flaccid paralysis (AFP) surveillance indicators in Guinea by region, 2012-2015: Non-polio acute flaccid paralysis (NP-AFP) rate

**Figure 2:**
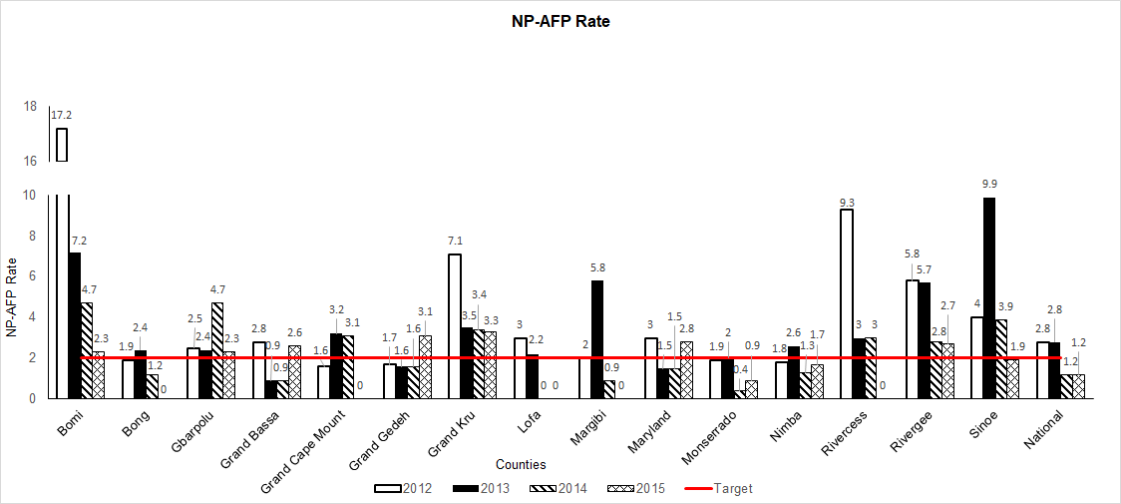
acute flaccid paralysis (AFP) surveillance indicators in Liberia by county, 2012-2015: non-polio acute flaccid paralysis (NP-AFP) rate; NOTE: NP-AFP rate is the number of non-polio AFP cases identified in population aged < 15 years per 100,000 population aged < 15 years

**Figure 3:**
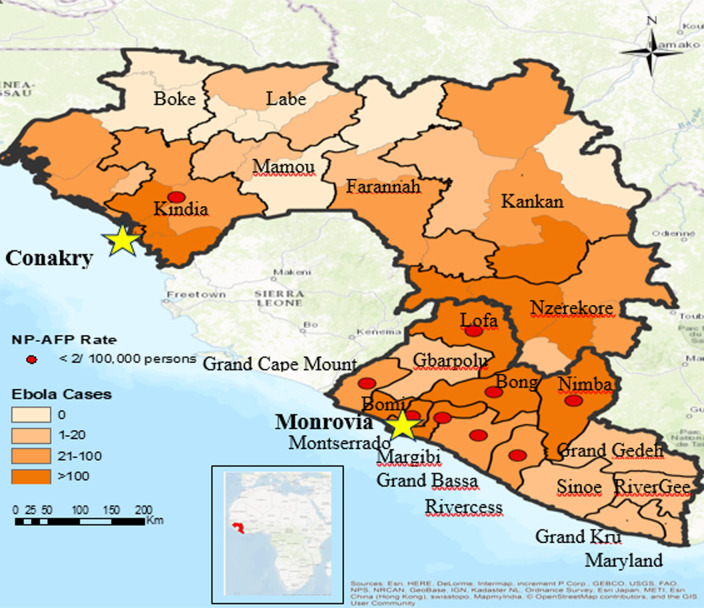
Ebola cases and detection of nonpolio-acute flaccid paralysis (NP- AFP) cases in Guinea & Liberia (2014 & 2015)

For Guinea, the national annual NP-AFP rates decreased from the pre-Ebola period (2012-2013) to the Ebola period (2014-2015); the rate dropped from 4.0 cases per 100,000 persons aged < 15 years in 2012 to 2.8 in 2015. Sub-nationally, the NP-AFP rate decreased from the pre-Ebola period to the Ebola period in seven of eight regions; during the Ebola period, the NP-AFP rate fell below the minimal standard of 2.0 cases per 100,000 population aged < 15 years in two regions (Conakry and Kindia) (Figure 1); Note: NP-AFP rate is the number of non-polio AFP cases identified in population aged < 15 years per 100,000 population aged < 15 years). In Liberia, the national NP-AFP rate dropped from 2.8 in 2012 to 1.2 in 2015 (Figure 2). On the sub-national level, the NP-AFP rate also decreased to fewer than 2.0 cases per 100,000 population aged < 15 years in both 2014 and 2015 in 11 of 15 counties (Bong, Grand Bassa, Grand Cape Mount, Grand Gedeh, Lofa, Margibi, Maryland, Montserrado, Nimba, Rivercess, and Sinoe) (Figure 2). No AFP cases were reported from Lofa County, the epicenter of Liberia's EVD outbreak, beginning in 2014, whereas several cases had been reported in the pre-Ebola period. As the Ebola outbreak progressed into 2015, the number of counties not reporting any AFP cases increased to five (Bong, Grand Cape Mount, Margibi, Lofa, and Rivercess). The regions of Guinea and counties of Liberia that experienced NP-AFP rates < 2.0 cases per 100,000 population aged < 15 years during the Ebola period were the geographical areas that experienced the highest total number of Ebola cases (Figure 3).

### Notification and proportion of AFP cases with adequate specimens

**Figure 4:**
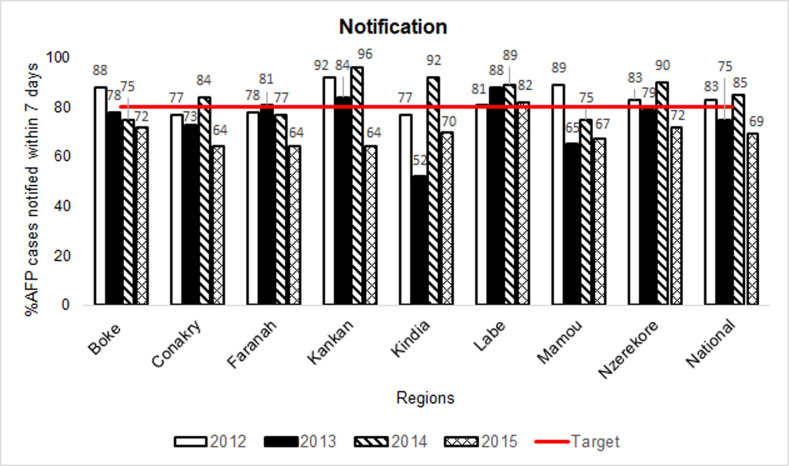
acute flaccid paralysis (AFP) surveillance indicators in Guinea by region, 2012-2015: notification (Note 1: notification is the proportion of AFP cases that were reported to the health system within 7 days of paralysis onset; Note 2: cases with missing data were deducted from the numerators and denominators for the calculation of the “notification” indicator)

**Figure 5:**
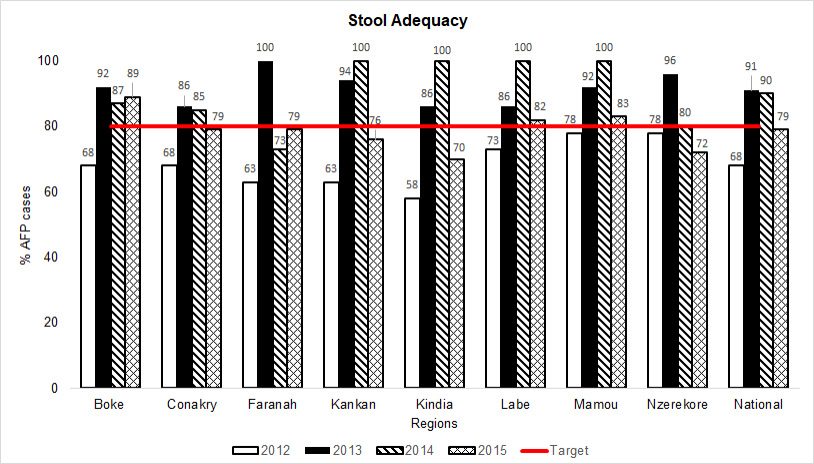
acute flaccid paralysis (AFP) surveillance indicators in Guinea by region, 2012-2015: stool adequacy (Note 1: stool adequacy is the number of AFP cases that had 2 stools collected within 14 days from paralysis onset and judged to be in good condition upon arrival at the laboratory; Note 2: cases with missing data were deducted from the numerators and denominators for the calculation of the “stool adequacy” indicator)

**Figure 6:**
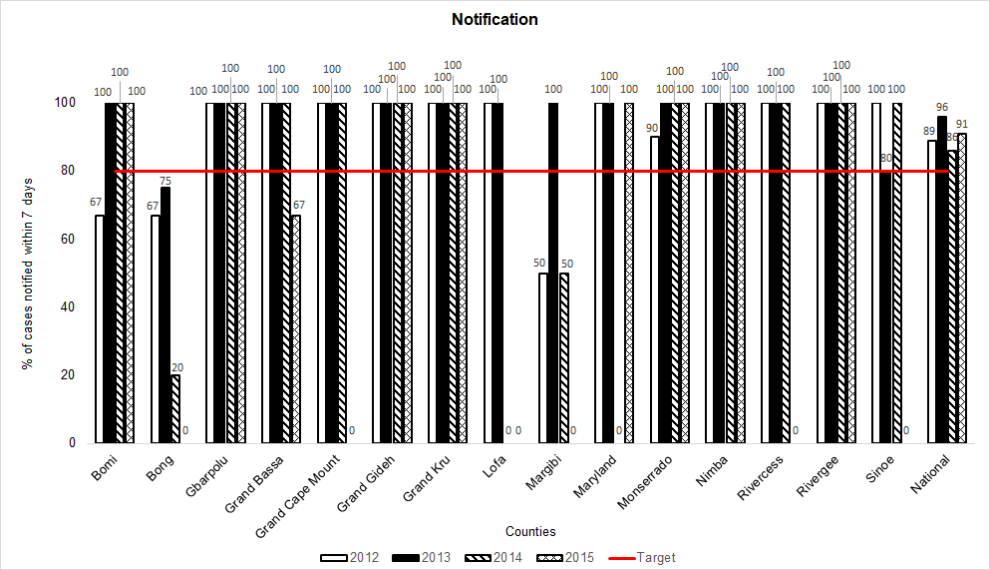
acute flaccid paralysis (AFP) surveillance indicators in Liberia by county, 2012-2015: notification (Note 1: notification is the proportion of AFP cases that were reported to the health system within 7 days of paralysis onset; Note 2: cases with missing data were deducted from the numerators and denominators for the calculation of the “notification” indicator)

**Figure 7:**
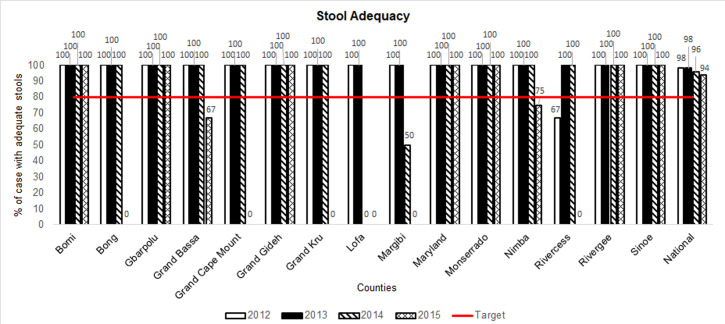
acute flaccid paralysis (AFP) surveillance indicators in Liberia by county, 2012-2015: stool adequacy (Note 1: stool Adequacy is the number of AFP cases that had 2 stools collected within 14 days from paralysis onset and judged to be in good condition upon arrival at the laboratory; Note 2: cases with missing data were deducted from the numerators and denominators for the calculation of the “stool adequacy” indicator)

Guinea inconsistently met the timely case notification threshold of 80% nationally throughout the study period, with seven of eight regions falling below this threshold in 2015 (Figure 4). The indicator for stool adequacy appeared to improve before the Ebola outbreak and through 2014, but fell below the 80% target in 2015 in five of eight counties (Figure 5). In Guinea, 39 AFP cases were missing a stool condition designation in 2014. In Liberia, for the 10 counties where AFP cases were reported, the surveillance indicators for notification and stool adequacy met or exceeded the 80% target in most counties across all time periods (Figure 6 and Figure 7).

## Discussion

AFP surveillance is one of the key strategies for polio eradication and the primary means of detecting poliovirus transmission. In some settings, testing of sewage samples (environmental surveillance) has supplemented AFP surveillance in identifying poliovirus circulation [17]. As long as WPV transmission continues, the risk of outbreaks in currently polio-free areas of the world will continue; populations in crisis due to armed conflict or other emergency conditions are especially vulnerable. In 2013, WPV that originated in Pakistan caused an outbreak in Syria and spread to Iraq due to the near collapse of the health systems caused by the ongoing armed conflict in the region; a strong, multi-country immunization response and an intensified AFP surveillance effort were mounted [33]. In Somalia, long-term civil unrest culminated in large immunization coverage gaps; after introduction of WPV from Nigeria, a large outbreak occurred in 2013 that spread into Kenya and Ethiopia [34]. During 2017-2018, cVDPV2 outbreaks occurred in Syria, the Democratic Republic of the Congo, Nigeria, and Somalia that required the use of monovalent OPV type 2 for response [17,35]. These outbreaks highlight the importance of maintaining high bOPV immunization levels, strong AFP surveillance to limit WPV transmission following importation or VDPV emergences and spread, as well as the multinational efforts needed to respond when an outbreak occurs.

Prior to the Ebola outbreak, the health systems of both Guinea and Liberia were already challenged by limited human resources and infrastructure [36]. Under typical conditions, the detection of WPV or VDPV cases leads to an intensification of AFP surveillance to identify potentially linked cases. However, this was not the case in Guinea during the Ebola outbreak; the first VDPV2 case was detected in 2014, but the community investigation was not fully performed and surveillance was not intensified. Polio SIAs were implemented only after genetically-linked cVDPV2 cases were identified in September 2015. Under the declining surveillance conditions of the Ebola period, few AFP cases were detected in late 2014 and the early part of 2015 (data not shown); once SIAs and special field exercises in Kankan and Faranah regions were triggered, additional AFP cases were identified as public health staff members began active community case searching, going door to door.

Both Guinea and Liberia saw reductions in their overall national NP-AFP rates during the Ebola period compared with the previous two years pre-Ebola. However, despite these reductions, Guinea continued to document above-target NP-AFP rates throughout the study period in all but two regions. In Liberia, the population size of children younger than 15 years of age in 9 of 15 counties is fewer than 100,000, too low to expect two AFP cases each year; however, many of these smaller counties reported two or more AFP cases annually pre-Ebola. Nonetheless, during the pre-Ebola period, only four counties (26%) in Liberia failed to reach the target NP-AFP rate; during the Ebola period, the number increased to 10 counties (66%), including the larger counties of Bong, Lofa, and Margibi where the population of children aged < 15 years is well above 100,000 (Table 1). During the Ebola outbreak, stool collection, shipment, and processing were affected. In Guinea, 39 AFP cases were missing a stool condition designation in 2014. Due to a lack of shipping and laboratory testing protocols in place for the safe handling of possible Ebola-infected samples, it was difficult to find courier services that could deliver samples for testing and for laboratories to test shipped specimens. As a result, an unknown number of stool specimens from AFP patients were destroyed [37]. These issues resulted in a 6-month gap in 2015 during which no stool specimens were tested for poliovirus in Guinea [38].

In addition to the effect of the EVD outbreak on health services, fear of contracting Ebola at health facilities and diminished community trust of the health system caused reductions in overall health-care-seeking behavior [23-28]. These health system and community effects contributed to the diminished functioning of the AFP surveillance systems. With external assistance to help restore vigorous infectious disease surveillance, notification of AFP cases increased after the Ebola outbreak in both countries. Particularly because of the cVPDV2 outbreak response in Guinea, 1,298 AFP cases were reported in 2016, reflecting the increase in case detection, and sample collection increased as well [38]. AFP surveillance indicators for Guinea and Liberia met standards nationally and sub-nationally during 2016-2017 [17].

**Disclaimer:** the findings and conclusions in this report are those of the author(s) and do not necessarily represent the official position of the U.S. Centers for Disease Control and Prevention.

## Conclusion

The EVD outbreak negatively affected AFP surveillance performance in both Guinea and Liberia, particularly sub-nationally. During crises in the health-care system, AFP surveillance systems must be reinforced to ensure that they continue to function.

### What is known about this topic

- *Tremendous progress has been made in eradicating polio worldwide;*
- *Having a strong health system in place is indispensable in achieving Polio eradication;*
- *The 2014 EVD outbreak claimed the lives of many people in West Africa and left behind a fragile health system*.

### What this study adds

- *Describes the status of acute flaccid paralysis (AFP) surveillance at the decentralized level in Guinea and Liberia before and during the 2014-2015 Ebola outbreak;*
- *Assesses the status of AFP surveillance in regions that were most affected by the Ebola outbreak;*
- *Describes the negative impact of the Ebola outbreak on AFP surveillance and polio eradication efforts in Guinea and Liberia*.
